# Dynamic Bacterial Communities, Resistome–Virulome Coupling, and Biomonitoring Paradigms at Direct Sea Discharge Outlets: An Integrated Microbiome Perspective for Coastal Pollution Control

**DOI:** 10.3390/microorganisms14071401

**Published:** 2026-06-25

**Authors:** Bingkun Wang, Shulei Jia, Lingling Chen, Miming Zhang

**Affiliations:** 1 The Third Institute of Oceanography, Ministry of Natural Resources, Xiamen 361005, China; 2 School of Basic Medical Sciences, Tianjin Medical University, Tianjin 300070, China

**Keywords:** bacterial community dynamics, direct sea discharge outlet, antibiotic resistance, virulome, horizontal gene transfer

## Abstract

Direct sea discharge outlets served as critical conduits for urban sewage and industrial wastewater disposal, playing dual roles as pollutant dilution channels and hotspots for pathogens and antibiotic resistance genes. Traditional monitoring approaches relying on physicochemical parameters and fecal indicator bacteria failed to capture the latent and cumulative risks posed by complex microbial communities. In this review, a holistic microbiome perspective was adopted to systematically synthesize current knowledge on the bacterial community dynamics, assembly mechanisms, resistome–virulome coupling patterns, mobilome-associated risk characteristics, and emerging biomonitoring strategies in direct sea discharge outlets. By integrating high-throughput multi-omics technologies with ecological network analysis and machine learning, we delineated a paradigm shift from cataloging microbial presence to deciphering functional interactions, risk propagation dynamics, and proactive surveillance strategies. Furthermore, under the “One Health” framework, we discussed emerging research frontiers and future challenges in managing pollution at discharge outlets, aiming to provide a scientific basis for environmental risk management in coastal zones.

## 1. Introduction

Direct sea discharge outlets serve as critical infrastructure for coastal cities to discharge treated or untreated sewage into marine environments, leveraging the dilution and self-purification capacities of oceans to mitigate pollutant concentrations [1,2]. However, mounting evidence reveals that these outfalls function not only as conduits for chemical pollutants, such as heavy metals, nutrients, and emerging organic contaminants, but also as hotspots for microbial pollution, including pathogenic bacteria, antibiotic resistance genes (ARGs), and virulence factors (VFs) [3,4,5]. In this review, the term “direct sea discharge outlet” (hereafter referred to as “discharge outlet” or “outfall”) is used generically to describe any point-source coastal infrastructure, including submarine outfalls, marine outfalls, and sewage discharge pipes, that releases treated or untreated effluent directly into marine environments. The complexity of microbial communities and their associated resistomes in outfall-impacted coastal waters underscores a pressing environmental and public health challenge that transcends traditional water quality monitoring paradigms [6,7].

Conventional coastal water quality assessments primarily rely on physicochemical parameters, such as chemical oxygen demand and ammonia nitrogen, and fecal indicator bacteria (FIB) like *Escherichia coli* and *Enterococci* [8,9]. While these indicators provide valuable information on recent fecal contamination, they suffer from critical limitations. First, FIB exhibit ambiguous host specificity, making it difficult to distinguish human sewage from agricultural or wildlife sources [10,11]. Second, many pathogens enter a viable but non-culturable (VBNC) state in marine environments, leading to severe underestimation of their presence when using culture-based methods [12,13]. Third, FIB do not capture ARGs, VFs, or their mobilization potential, which are increasingly recognized as latent and cumulative risks [14,15]. For instance, studies in diverse coastal regions have isolated multidrug-resistant *E. coli*, *Klebsiella* spp., *Enterococcus faecalis*, and *Vibrio cholerae* from sewage discharge points, with isolates frequently carrying clinically relevant ARGs such as *bla*_CTX-M_ and *vanA* [16,17,18]. These findings underscore the inability of traditional indicators to capture the full spectrum of microbial hazards posed by outfalls.

Wastewater treatment plants (WWTPs), while reducing overall bacterial loads, often serve as reservoirs and dissemination points for ARGs and multidrug-resistant bacteria [19,20]. Metagenomic and 16S rRNA amplicon sequencing analyses of effluent-receiving waters have revealed significant alterations in bacterial community composition, characterized by enrichment of non-indigenous taxa such as *Acinetobacter*, *Pseudomonas*, and *Salmonella* spp., which carry diverse ARGs conferring resistance to β-lactams, tetracyclines, macrolides, and multidrug efflux pumps [21,22,23]. Moreover, the persistence of clinically relevant vancomycin-resistant *Enterococcus faecium* strains in both treated and untreated wastewater, coastal bathing waters, and hospital effluents underscores the potential for sewage-driven dissemination of high-risk pathogens into marine environments [16]. Beyond bacterial pathogens, outfalls contribute to the release of microplastics and chemical pollutants such as phthalate esters and bisphenol A, which act as vectors and reservoirs for multidrug-resistant bacteria [24,25]. Experimental studies have demonstrated colonization of microplastics by carbapenem-resistant Enterobacteriaceae, highlighting the role of microplastics in promoting persistence and dissemination of antimicrobial resistance (AMR) in coastal waters [24,26].

The dynamics of ARGs in outfall-influenced coastal environments are shaped by multiple interacting factors, including horizontal gene transfer (HGT), microbial community interactions, and environmental conditions such as temperature, salinity, and nutrient availability [27,28,29]. Studies simulating sewage influx into coastal waters have shown that suspended particles mediate delayed decay of ARGs compared to free-living fractions, facilitating prolonged persistence and potential dissemination of resistance determinants [27]. Residual disinfectants like chlorine, increasingly used during events such as the COVID-19 pandemic, can induce shifts in microbial community structure and ARG composition, promoting co-selection of biocide resistance genes and ARGs [30]. Heavy metals, frequently detected in outfall sediments, exert co-selective pressures that enrich bacteria harboring both metal resistance genes and ARGs on the same mobile genetic elements (MGEs) [31,32]. Despite these findings, most studies have examined ARGs and VFs separately, leaving the coupling patterns between resistome and virulome and their co-dissemination mechanisms largely unexplored [21,33]. Furthermore, the relative contributions of deterministic (environmental filtering) versus stochastic (dispersal limitation, drift) processes in shaping outfall bacterial communities remain debated, with limited integration of null model analyses [34,35].

Several critical knowledge gaps persist. First, the physical co-localization of ARGs and VFs on conjugative plasmids or integrons has been demonstrated in clinical settings, but its prevalence and ecological drivers in marine outfall environments are poorly quantified [36]. Second, the role of bacteriophages in transducing ARGs and VFs in coastal waters is contested, with conflicting evidence from metaviromic studies [37,38]. Third, emerging biomonitoring tools such as the Microbiome Deviance Index (MDI) and the Antibiotic Resistance Risk Index (ARRI) have been proposed but lack validation in dynamic outfall systems [39,40]. Fourth, the “One Health” transmission pathways from outfalls through marine food webs to humans remain largely inferential, with few quantitative microbial risk assessment (QMRA) studies specific to direct sea discharges [41]. Finally, the detection and risk characterization of VBNC pathogens and “dark matter” ARGs represent major methodological frontiers [42,43].

Given these multifaceted challenges, there is an urgent need to transcend traditional monitoring approaches and adopt holistic, microbiome-based frameworks. This review distinguishes itself from prior works by: (i) systematically synthesizing the coupled resistome–virulome dynamics in outfall environments, including co-occurrence patterns, co-selection mechanisms, and mobilome-driven HGT; (ii) integrating ecological assembly theory (deterministic vs. stochastic processes) with pollution gradient analyses and temporal dynamics, including extreme events; (iii) critically evaluating emerging biomonitoring paradigms (MDI, ARRI, eDNA-microfluidics) and comparing their strengths, limitations, and validation status against traditional indicators; (iv) providing a forward-looking perspective on artificial intelligence, digital twins, and unresolved challenges (VBNC pathogens, resistance dark matter, phage transduction) within the One Health framework.

To achieve these goals, we first summarize advanced methodologies, followed by an examination of bacterial diversity and community assembly processes under pollution gradients. We then focus on the coupling of resistome and virulome, co-selection mechanisms, and the role of the mobilome. The discussion transitions from indicator organisms to integrated biological monitoring, encompassing microbiome-level indices and the potential of eDNA. Finally, we address One Health transmission pathways, virus–bacteria interactions, AI/digital twins, and unresolved issues, before concluding with a forward-looking perspective. By integrating high-throughput multi-omics with ecological network analysis and machine learning, this review delineates a paradigm shift from cataloging microbial presence to deciphering functional interactions, risk propagation dynamics, and proactive surveillance strategies for direct sea discharge outlets.

## 2. Advanced Methodologies

### 2.1. High-Throughput Multi-Omics Technologies

High-throughput multi-omics technologies have revolutionized the study of microbial communities in marine outfall environments by enabling comprehensive characterization of microbial diversity, functional potential, and activity. Amplicon sequencing targeting marker genes such as 16S rRNA for bacteria, 18S rRNA for eukaryotes, and ITS regions for fungi remains the foundational approach to delineate the taxonomic composition of microbial communities at high resolution. This method allows identification of dominant taxa as well as rare biosphere members, providing insights into community structure and potential ecological roles within the outfall microbiome. For instance, 16S rRNA gene sequencing has been widely applied to distinguish free-living, particle-attached, and biofilm-associated bacterial communities in coastal waters, revealing lifestyle-specific assemblages influenced by pollution gradients and environmental parameters [44]. However, amplicon sequencing is limited to taxonomic profiling and cannot directly assess functional genes or their expression.

To overcome this limitation, metagenomic sequencing offers an unbiased, genome-resolved view of the entire microbial community, capturing the full repertoire of ARGs, VFs, and mobile genetic elements present in the microbiome. This approach enables detection of the resistome and virulome within the outfall environment, critical for understanding the co-occurrence and potential horizontal transfer of resistance and virulence determinants. For example, metagenomic analyses of marine Enterobacterales plasmidomes have identified clinically relevant ARGs such as *bla*_KPC_ and *bla*_TEM_ co-localized with insertion sequences and broad-host-range plasmids, highlighting the role of coastal pollution in shaping resistance gene dissemination [45]. Metagenomics also supports assembly-based functional annotation, allowing prediction of metabolic pathways and pollutant degradation capabilities, thus linking microbial community composition to ecosystem functions.

Beyond short-read metagenomics, the application of third-generation sequencing technologies, such as PacBio HiFi and Oxford Nanopore, has significantly advanced the resolution of resistome and virulome characterization in direct sea outfall environments. By generating long contiguous reads, these platforms overcome limitations in assembling repetitive genomic regions, such as insertion sequences and integron cassettes, that frequently harbor ARGs and VFs. For example, Nanopore sequencing has facilitated the recovery of complete plasmid sequences from complex marine metagenomes, enabling precise determination of ARG-VFs co-localization and mobile genetic elements (MGEs) architecture without assembly gaps [45]. Additionally, long-read sequencing provides high-resolution insights into the host taxonomy of MGEs through direct read-level assignment, which is often obscured in short-read assemblies due to genomic similarity across taxa. However, challenges such as high DNA input requirements, elevated sequencing error rates relative to short-read platforms, and the need for optimized bioinformatic pipelines tailored to complex environmental matrices limit routine application. Complementary to sequencing innovations, single-cell genomics and high-throughput culturomics are emerging as powerful tools to link functional gene identity directly to host cells. Single-cell techniques bypass the need for cultivation by isolating individual bacterial cells and amplifying their genomes, thus revealing the genetic repertoire of uncultured majority taxa. In direct sea outfall sediments, single-cell approaches have uncovered novel ARG-host associations that were not detected via bulk metagenomic analyses, expanding our understanding of the phylogenetic distribution of resistance determinants. Culturomics, on the other hand, couples high-throughput cultivation conditions with MALDI-TOF mass spectrometry or 16S rRNA gene sequencing to capture previously uncultured species. Applied to outfall-impacted sediments, culturomics has recovered clinically relevant multidrug-resistant isolates belonging to the ESKAPEE group, providing valuable phenotypic context to metagenomic predictions. Integrating these targeted methodologies with broad-scale multi-omics creates a complementary pipeline that bridges the resolution gap between gene detection and functional host identification.

Complementing metagenomics, metatranscriptomics provides a dynamic perspective by profiling the active gene expression of microbial communities under environmental stressors such as pollution. By analyzing RNA transcripts, metatranscriptomics reveals which ARGs and VFs are actively transcribed, distinguishing between latent genetic potential and real-time functional risk. This distinction is crucial for risk assessment, as the presence of resistance genes does not necessarily imply their expression or impact. Studies on oyster gut microbiomes along eutrophication gradients demonstrated that while microbial taxa remained relatively stable, gene expression related to nutrient cycling varied with local environmental conditions, reflecting functional plasticity in response to pollution stress [46]. Similarly, metatranscriptomic data can identify transcriptional activation of virulence factors in pathogenic bacteria such as *Vibrio fortis* during coral infection, linking microbial shifts to disease outcomes [47]. A critical comparison of these omics approaches reveals distinct trade-offs. Amplicon sequencing is cost-effective and computationally straightforward, making it suitable for large-scale surveys; however, it provides taxonomic information only and cannot assess functional genes [44]. Metagenomic sequencing delivers comprehensive functional profiles (resistome, virulome, mobilome) but requires higher sequencing depth and sophisticated bioinformatics [45]. Metatranscriptomics distinguishes active expression from latent potential, yet RNA instability and high rRNA background pose challenges [46,47]. Third-generation long-read sequencing (PacBio, Oxford Nanopore) resolves repetitive elements and enables complete plasmid assembly, but exhibits higher per-base error rates and demands high-molecular-weight DNA [45]. Single-cell genomics and culturomics can link genes to host cells but are low-throughput and technically demanding [48,49]. Consequently, a tiered approach is recommended: amplicon screening for initial profiling, followed by targeted metagenomics for ARG/VF detection, and metatranscriptomics for mechanistic studies. Thus, integrating amplicon sequencing, metagenomics, and metatranscriptomics provides a holistic multi-omics framework to unravel the complex dynamics of microbial communities, resistomes, and virulomes in direct sea outfall environments, enabling precise monitoring and mechanistic understanding of pollution impacts.

### 2.2. Quantitative and Source-Tracing Tools

Accurate quantification and source attribution of ARGs, VFs, and pathogenic markers in coastal outfall environments are essential for pollution control and risk management. Droplet digital PCR has emerged as a powerful quantitative tool that overcomes the limitations of traditional quantitative PCR, such as dependence on standard curves and susceptibility to PCR inhibitors. ddPCR partitions the PCR reaction into thousands of nanoliter droplets, enabling absolute quantification of target genes with high sensitivity and precision, particularly for low-abundance targets typical in environmental samples. This technology has been successfully applied to quantify ARGs and VFs in sewage-impacted coastal waters, providing robust data for assessing pollution levels and temporal trends [17]. Its ability to detect subtle changes in gene abundance facilitates early warning of emerging resistance threats and evaluation of remediation efficacy.

Microbial source tracking complements quantitative approaches by identifying the origins of fecal contamination in coastal outfalls, a critical step for targeted pollution mitigation. Microbial source tracking (MST) employs host-specific genetic markers derived from obligate anaerobic bacteria such as Bacteroides and Bifidobacterium, which exhibit high host specificity to humans, livestock, or wildlife. By detecting these markers, MST can precisely differentiate human sewage inputs from agricultural runoff or wildlife sources, resolving the complex mixture of fecal pollution in marine environments. For example, MST markers have been used to distinguish human-derived fecal contamination in Indian coastal waters, correlating with the presence of antibiotic-resistant pathogens and informing public health interventions [17]. The integration of MST with quantitative gene detection enables source-resolved risk assessment, guiding regulatory actions and infrastructure improvements to reduce pollutant loads at their origin.

Together, ddPCR and MST form a complementary toolkit for absolute quantification and precise source attribution of microbial pollutants in direct sea outfall environments. These tools enhance the resolution and reliability of microbial pollution monitoring, enabling differentiation of anthropogenic impacts from natural background levels. Their application supports evidence-based coastal pollution control strategies by identifying priority sources, quantifying pollutant loads, and tracking the effectiveness of management interventions over time.

### 2.3. Ecological Networks and Machine Learning

Machine learning models such as Random Forest (RF) and SourceTracker leverage multivariate environmental data (temperature, salinity, pollutant concentrations, nutrients) to predict ARG abundance and distribution patterns in coastal outfalls [50]. RF, an ensemble learning method based on decision trees, effectively captures non-linear relationships and interactions among predictors. Its implementation involves training on feature matrices with cross-validation to prevent overfitting and hyperparameter tuning using grid search or Bayesian optimization. Key implementation challenges include: (i) the “curse of dimensionality” when features greatly exceed sample size; (ii) data heterogeneity across studies due to different sequencing platforms; (iii) the need for large, standardized training datasets; and (iv) limited model interpretability, although SHAP values can partially address this. For source apportionment, SourceTracker employs a Bayesian Dirichlet-multinomial model to estimate proportional contributions of potential pollution sources to a sink community [17]. Its strength lies in probabilistic source attribution, but it assumes that source communities are well-characterized and that no unknown sources exist. Recent advances integrating RF with teaching-learning-based optimization algorithms have enhanced coastal water quality prediction accuracy [50]. Overall, combining RF for hotspot prediction and SourceTracker for source attribution provides a complementary ML toolkit, yet standardized benchmarking and open-access training datasets are urgently needed.

## 3. Bacterial Diversity and Community Construction in Direct Sea Discharge Outlets

### 3.1. Diversity Patterns Under Pollution Gradients

**Figure 1:**
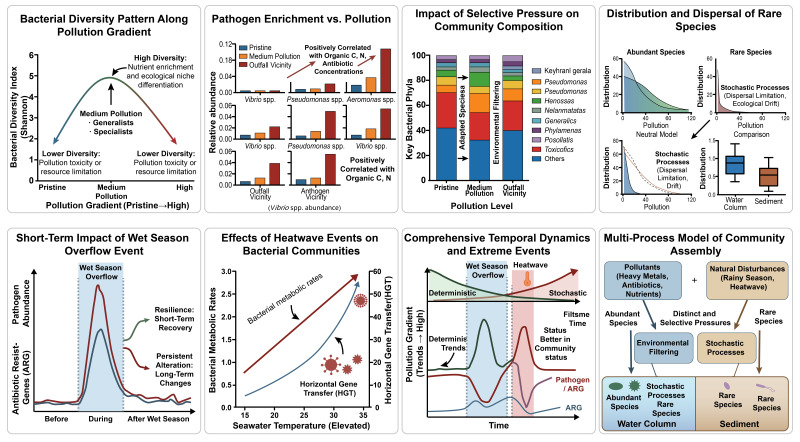
Schematic overview of bacterial community diversity patterns, assembly mechanisms, and temporal dynamics in direct sea discharge outlet environments.

The diversity patterns of bacterial communities along pollution gradients in marine outfall environments reveal critical insights into ecosystem responses to anthropogenic disturbances. The intermediate disturbance hypothesis is strongly supported in these contexts, where bacterial diversity peaks at moderate pollution levels. The phenomenon is attributed to the balance between nutrient enrichment and environmental stress, which fosters niche differentiation and coexistence of diverse taxa. For instance, studies in estuarine systems such as the Bay of Bengal demonstrate that moderate sewage pollution and nutrient inputs elevate bacterial diversity, as moderate organic carbon and nitrogen concentrations create favorable conditions for a wide range of bacteria, including both generalists and specialists [51]. This peak in diversity at intermediate disturbance levels contrasts with lower diversity observed in heavily polluted or pristine sites, where either toxic stress or resource limitation restricts community complexity (Figure 1).

In the near-field regions of marine outfalls, opportunistic pathogenic bacteria such as *Vibrio* spp., *Pseudomonas* spp., and *Aeromonas* spp. are significantly enriched. Their abundance correlates positively with organic carbon, nitrogen, and antibiotic concentrations, indicating that these pollutants provide selective advantages for these taxa. For instance, *Aeromonas* and *Vibrio* species, known for their pathogenic potential in aquatic organisms and humans, thrive in nutrient-rich and antibiotic-contaminated waters near sewage discharge points [48,49]. The co-occurrence of these pathogens with elevated antibiotic residues suggests that outfall environments act as hotspots for the proliferation and potential dissemination of antibiotic-resistant bacteria. Moreover, the presence of these opportunistic pathogens is not only linked to nutrient enrichment but also to environmental parameters such as temperature and salinity, which modulate their growth and virulence expression [51].

The enrichment of these pathogens in outfall vicinities underscores the dual role of pollution as both a driver of microbial diversity and a selective pressure favoring potentially harmful bacteria. This dynamic has important implications for coastal pollution control, as it highlights the need to monitor not only chemical contaminants but also microbial community shifts that may pose risks to ecosystems and human health. The observed diversity patterns suggest that managing pollution inputs to maintain moderate disturbance levels could help preserve microbial diversity while limiting the dominance of opportunistic pathogens. However, given the complexity of interactions among nutrients, pollutants, and microbial taxa, integrated microbiome-based monitoring approaches are essential for effective coastal pollution management (Figure 1).

### 3.2. Community Assembly Processes

The assembly of bacterial communities in marine outfall environments is governed by a combination of deterministic and stochastic processes, as revealed by neutral and null model analyses. Deterministic processes, primarily environmental filtering, play a dominant role in shaping the composition of abundant taxa by selecting for species adapted to gradients of heavy metals, antibiotics, and nutrient concentrations. For example, the presence of heavy metals such as copper and mercury, alongside antibiotic residues, imposes strong selective pressures that favor resistant and tolerant bacterial groups, including the *Aeromonas* and *Pseudomonas* species [18,52]. These pollutants act as environmental filters that exclude sensitive taxa and promote the proliferation of resistant strains, thereby structuring the core microbiome of the outfall vicinity (Figure 1). In submarine outfall sediments co-contaminated with copper (Cu, 45–120 mg/kg) and mercury (Hg, 0.3–2.1 mg/kg), variable selection accounted for over 60% of phylogenetic turnover in abundant bacterial taxa, as quantified by the Stegen null model [18]. The normalized stochasticity ratio (NST) decreased from 0.68 in reference sites to 0.29 in high-pollution zones, indicating a shift from stochastic to deterministic assembly [34].

In contrast, stochastic processes such as dispersal limitation and ecological drift are more influential in the rare biosphere, contributing to the presence and dynamics of low-abundance taxa. The neutral community model (NCM) explained 72–85% of the distribution of rare taxa in the water column (immigration rate Nm = 12–18), but only 45–58% in benthic sediments, suggesting stronger dispersal limitation in the benthos [34]. These random processes introduce variability in community composition, especially in less polluted or transitional zones where environmental filtering is weaker. The interplay between deterministic and stochastic forces results in spatial heterogeneity and temporal fluctuations in bacterial assemblages, reflecting the complex ecological landscape of marine outfall systems [34]. Advancing beyond qualitative identification of deterministic versus stochastic contributions, recent quantitative frameworks utilizing the Stegen null model and Normalized Stochasticity Ratio (NST) provide more nuanced insights. By parsing phylogenetic turnover into selection, dispersal limitation, homogenizing dispersal, and ecological drift, these models reveal that pollutant loading significantly elevates the relative importance of variable selection on abundant taxa [34,35]. For instance, in outfall sediments exhibiting metal and antibiotic co-contamination, variable selection can account for over 60% of phylogenetic turnover [53]. Complementing phylogenetic approaches, the neutral community model (NCM) estimates immigration rate (m) and the fit of stochastic dynamics for the rare biosphere. Studies in urbanized estuaries indicate that the neutral model consistently explains a higher proportion of rare taxa distribution in water columns (~70–85%) compared to sediments (~40–60%), underscoring that hydrodynamic mixing promotes random dispersal in the planktonic fraction whereas benthic habitats experience stronger deterministic selection [34,51]. Furthermore, coupling water and sediment compartments represents a critical frontier. Particle settling and sediment resuspension create a bidirectional microbial loop. Assembly models that concurrently evaluate both compartments suggest that deterministic selection strength decays along the water-sediment continuum during stratified conditions, but storm events synchronize assembly processes through enhanced mass effects [35]. Integrating these compartmental assembly models with longitudinal sampling is essential for predicting microbial responses to both chronic pollution and episodic disturbances.

Advancing beyond the qualitative identification of deterministic and stochastic contributions, recent quantitative frameworks utilizing the Stegen null model and Normalized Stochasticity Ratio have provided more nuanced insights into community assembly. By parsing phylogenetic turnover into selection, dispersal limitation, homogenizing dispersal, and ecological drift, these models reveal that pollutant loading significantly elevates the relative importance of variable selection on abundant taxa. For instance, in submarine outfall sediments exhibiting metal and antibiotic co-contamination, variable selection can account for over 60% of phylogenetic turnover, demonstrating that harsh geochemical gradients act as deterministic sieves. Complementing phylogenetic-based approaches, the neutral community model (NCM) has been employed to estimate the immigration rate and the fit of stochastic birth-death dynamics for the rare biosphere. Studies in urbanized estuaries receiving outfall effluents indicate that the neutral model consistently explains a higher proportion of rare taxa distribution in water columns compared to sediments, underscoring that hydrodynamic mixing promotes random dispersal in the planktonic fraction whereas benthic habitats experience stronger deterministic selection. Furthermore, the coupling between water and sediment compartments represents a critical yet underexplored frontier in community assembly research (Figure 1). Particle settling and sediment resuspension events create a bidirectional microbial loop wherein sediment-associated bacteria seed the water column and vice versa. Assembly models that concurrently evaluate both compartments suggest that deterministic selection strength decays along the water–sediment continuum during stratified conditions, but that storm events synchronize assembly processes, homogenizing community composition through enhanced mass effects. Integrating these compartmental assembly models with longitudinal sampling is essential for predicting how microbial community structure responds to both chronic pollution and episodic disturbances.

Co-contamination by heavy metals and antibiotics exerts synergistic selective pressures that enhance the fitness of antibiotic-resistant bacteria. This dual stress environment promotes the evolution and maintenance of resistance determinants, including carbapenemase genes and heavy metal resistance operons, often co-located on mobile genetic elements such as plasmids and transposons [18,32]. The co-selection mechanism promotes the persistence and dissemination of multidrug-resistant bacteria in outfall sediments and waters, thereby enriching the resistome. Consequently, the functional structure of bacterial communities shifts towards enhanced resistance capabilities, which may compromise ecosystem functions and pose public health risks. Overall, the community assembly in marine outfall environments reflects a balance between selective pressures imposed by pollutants and stochastic ecological processes. Elucidating these mechanisms is key to forecasting how microbial communities respond to pollution and to developing targeted strategies that limit the proliferation of resistance and pathogenic traits in coastal environments (Figure 1). Co-selection indices ranged from 0.34 to 0.67 in outfall-affected sediments, with significant positive correlations (Spearman’s *ρ* > 0.5, *p* < 0.01) between mercury resistance genes (*merA*) and β-lactamase genes (*bla*_TEM_) [32].

### 3.3. Temporal Dynamics and Extreme Events

Temporal dynamics of bacterial communities in marine outfall environments are profoundly influenced by episodic extreme events such as rain-induced overflows and heatwaves, which induce rapid and significant perturbations. Rainy season overflow events introduce high loads of untreated sewage and surface runoff into outfall vicinities, causing abrupt shifts in bacterial community structure. During rain-induced overflow events (precipitation > 50 mm/day), concentrations of *E. coli* and *Enterococci* in outfall-affected waters increased from background levels of ~10^2^ CFU/100 mL to >10^4^ CFU/100 mL, remaining elevated for 48–72 h [35]. Similarly, ARG copy numbers measured by ddPCR rose by 1.5–2.5 log10 copies/L within 6 h of overflow onset [54]. These inputs lead to transient surges in the abundance of pathogenic bacteria and ARGs, as observed in riverine and estuarine systems impacted by urban wastewater [35,54]. The magnitude and duration of these perturbations depend on the intensity and persistence of overflow events, as well as the baseline environmental conditions. While bacterial communities often exhibit resilience, characterized by recovery towards pre-disturbance states, prolonged or repeated overflows can lead to lasting alterations in community composition and function. In a 12-month monitoring study across water depths of 2–15 m, the most significant shifts in pathogen abundance (*Vibrio* spp., up to 3.2 × 10^3^ copies/L) occurred in the upper 5 m layer during summer months (June–August), coinciding with water temperatures >25 °C [51].

Heatwave events exacerbate microbial risks by elevating seawater temperatures, which accelerate bacterial metabolism and horizontal gene transfer rates. Heatwave conditions (seawater temperature increase from 22–28 °C over 5 days) enhanced conjugation transfer frequency of ARG-carrying plasmids from 1.2 × 10^−5^ to 8.7 × 10^−5^ transconjugants per recipient, a 7-fold increase [55]. Elevated temperatures enhance the expression of virulence factors in opportunistic pathogens such as *Vibrio* spp., increasing their pathogenic potential [51,55]. The metabolic acceleration under thermal stress also facilitates the proliferation of antibiotic-resistant and virulent strains, thereby amplifying the risk of disease outbreaks in marine organisms and humans. These temperature-driven dynamics underscore the vulnerability of outfall microbiomes to climate change-induced extreme events (Figure 1).

The combined effects of overflow and heatwave events highlight the importance of temporal monitoring to capture the episodic nature of microbial risks in marine outfall environments. The resilience of bacterial communities is contingent upon the frequency and severity of disturbances, as well as the capacity of the ecosystem to buffer pollutant inputs. Integrating temporal dynamics into microbial risk assessments and pollution control strategies is essential for safeguarding coastal ecosystem health and mitigating public health threats under changing climatic conditions (Figure 1; Table 1).

## 4. Coupling of Drug-Resistant, Virulence and Risk of Mobile Groups

### 4.1. Co-Occurrence Patterns of ARGs and VFs

**Figure 2:**
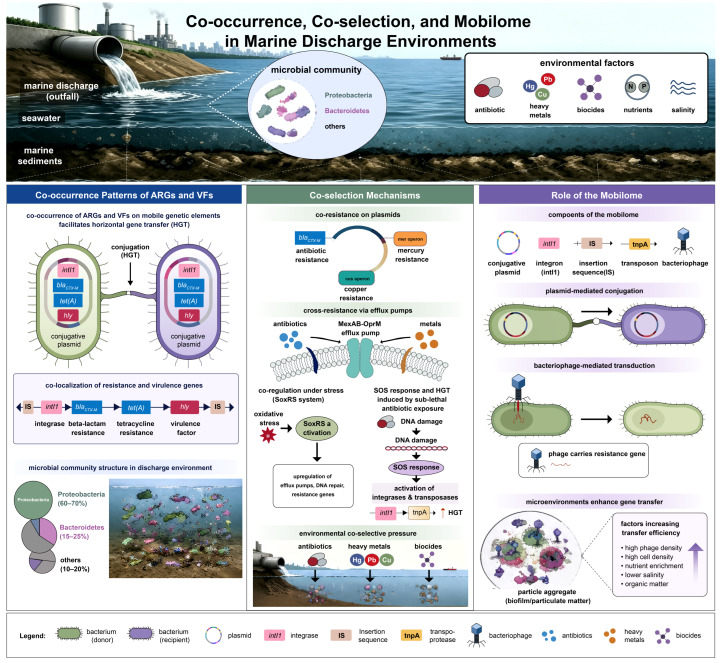
Conceptual model illustrating the coupling of antibiotic resistance and virulence genes, co-selection by metals and biocides, and mobilome-driven horizontal gene transfer in marine outfall ecosystems.

Statistical co-occurrence analyses of direct sea discharge outlet sediment and seawater samples consistently reveal a significant positive correlation between the abundance of ARGs and VFs, suggesting that these genetic elements may be co-carried by the same bacterial hosts or enriched synergistically within shared ecological niches. For instance, studies in coastal and estuarine environments have demonstrated that ARGs such as *sul1*, *tet*(*A*), and *bla*_CTX-M_ variants frequently co-occur with integrase genes like *intI1*, which are often linked to virulence determinants, indicating a coupled dissemination mechanism driven by MGEs [21,29]. Metagenomic assemblies and plasmid sequencing further substantiate the physical co-localization of ARGs and VFs on conjugative plasmids. Notably, plasmids harboring *bla*_CTX-M_ genes encoding β-lactam resistance alongside *hly* genes encoding hemolysin virulence factors have been repeatedly isolated from sediment samples at marine discharge points, underscoring the existence of “resistance-virulence” conjugative plasmids that significantly enhance bacterial pathogenic potential and horizontal transmission risk [33,36]. This co-localization facilitates the simultaneous spread of antimicrobial resistance and virulence traits, posing a compounded threat to marine ecosystem health and human exposure through recreational or seafood consumption pathways. Moreover, the co-enrichment of ARGs and VFs is influenced by anthropogenic pollution inputs, such as sewage and aquaculture effluents, which introduce both resistant and pathogenic bacteria into marine environments, thereby shaping the resistome and virulome profiles in discharge sediments and adjacent waters [22,57]. The ecological interplay between ARGs and VFs is further modulated by microbial community structure, with dominant phyla such as Proteobacteria and Bacteroidetes serving as key reservoirs for these genes, as evidenced by positive correlations between their abundance and ARG/VFs levels in marine sediments [21,58]. Collectively, these findings highlight the intricate genetic and ecological coupling of antibiotic resistance and virulence determinants in marine discharge environments, emphasizing the need for integrated surveillance strategies that consider both ARGs and VFs to effectively assess and mitigate public health risks associated with coastal pollution (Figure 2).

### 4.2. Co-Selection Mechanisms

Co-selection mechanisms play a pivotal role in driving the dissemination and persistence of ARGs in marine discharge environments, particularly through the interplay of heavy metals and biocidal compounds. Heavy metals such as copper and mercury, commonly detected in estuarine and coastal sediments impacted by anthropogenic activities, exert selective pressure that enriches bacteria harboring both metal resistance genes and ARGs on the same MGEs, such as plasmids and integrons (Figure 2). This co-resistance phenomenon enables bacteria to survive in metal-contaminated environments while concurrently maintaining antibiotic resistance, thereby indirectly promoting ARG proliferation even in the absence of antibiotic exposure [29,31,59].

At the molecular level, co-selection is mechanistically underpinned by several distinct genetic architectures. The most direct mechanism, co-resistance, occurs when ARGs and metal resistance genes are physically linked on the same MGE, as exemplified by the frequent co-localization of the mer operon (mercury resistance) with *bla*_NDM_ or *sul1* on class 1 integrons. Cross-resistance, whereby a single efflux pump such as MexAB-OprM in *Pseudomonas aeruginosa* exports both antibiotics and heavy metals, represents another critical pathway, enabling broad-spectrum survival under multi-stressor conditions. Additionally, co-regulation, in which a shared regulatory protein responds to either metal or antibiotic stress to simultaneously upregulate both resistance systems, has been documented in *E*. *coli* through the SoxRS regulation. These molecular mechanisms are not mutually exclusive but rather operate synergistically to stabilize multidrug-resistant phenotypes in outfall environments. Beyond toxic metals, the contribution of sub-inhibitory antibiotic and biocide concentrations to co-selection extends to the activation of the SOS response and reactive oxygen species pathways. At discharge outlets, antibiotic gradients generated by effluent dilution create microzones where concentrations fall below minimum inhibitory thresholds, acting as signaling molecules that induce the LexA/RecA-mediated SOS cascade (Figure 2). This response upregulates integrases and transposases, enhancing horizontal gene transfer (HGT) rates and promoting cassette excision and integration within integrons [60]. Simultaneously, ROS generation under combined antibiotic and metal stress induces oxidative damage that can increase membrane permeability and plasmid uptake competence, further accelerating ARG dissemination. Elucidating these intracellular signaling pathways and their environmental triggers is essential for identifying the molecular determinants of HGT hotspots (Figure 2). The co-selection driven by heavy metals and biocides thus extends beyond simple enrichment to encompass a complex regulatory network that amplifies the risk of ARG proliferation in coastal discharge zones.

Additionally, biocidal agents like triclosan, widely used as antimicrobial additives, have been implicated in co-selecting for ARGs by exerting cross-resistance or co-resistance effects, further complicating resistance dynamics in marine ecosystems [59]. The discharge outlet zones often act as “hotspots” of sub-inhibitory antibiotic concentrations, which induce bacterial stress responses such as the SOS response. This response activates the expression of integrases and transposases, enzymes critical for HGT, thereby enhancing the mobilization and spread of ARGs via conjugative plasmids and integrons [57,60]. Empirical evidence from estuarine and coastal studies demonstrates that environmental factors, including nutrient levels, salinity gradients, and pollutant loads, modulate the frequency of HGT events, with integron-integrase gene *intI1* serving as a key marker of such genetic exchange processes [28,61]. The co-selection driven by heavy metals and biocides thus creates a selective landscape that favors multidrug-resistant bacteria, amplifying the risk of ARG dissemination into marine food webs and human populations (Figure 2).

### 4.3. Role of the Mobilome

The mobilome, encompassing integrons, insertion sequences, plasmids, and bacteriophages, constitutes the central genetic framework facilitating the dissemination of ARGs and VFs in marine discharge environments. Class 1 integrons, characterized by the integrase gene *intI1*, are particularly prevalent in estuarine and coastal sediments, where they capture and express diverse gene cassettes encoding resistance determinants, thereby enabling rapid bacterial adaptation to environmental stressors including antibiotics and heavy metals [21,28]. Insertion sequences further contribute to genomic plasticity by mediating gene mobilization and rearrangements that enhance ARG propagation. Plasmids such as IncQ and RP4 have been identified as key vectors carrying both ARGs and VFs, with metagenomic analyses revealing their widespread presence in direct sea and coastal marine microbiomes [37,62]. The conjugative nature of these plasmids facilitates HGT across diverse bacterial taxa, amplifying the spread of multidrug resistance and virulence traits. The role of bacteriophages in mediating ARG and VFs transfer via transduction remains a subject of ongoing debate. While some studies have detected virulence-associated genes within marine viral metagenomes from discharge zones, direct evidence of phage-mediated transduction events is limited, necessitating further investigation combining metaviromics and culture-based assays to elucidate their contribution to gene flow [37,38]. Notably, the dynamics of the mobilome are influenced by environmental factors such as pollutant concentrations and microbial community composition, which modulate the frequency and efficiency of HGT. The enrichment of MGEs in discharge sediments and waters underscores their critical role in shaping the resistome and virulome landscapes in marine ecosystems impacted by anthropogenic pollution (Figure 2). Comprehensive characterization of the mobilome is thus imperative for understanding the mechanisms underpinning ARG and VFs dissemination and for informing effective biocontrol and monitoring strategies in coastal pollution management.

To resolve the long-debated contribution of phage-mediated transduction to ARG and VF dissemination, recent studies have employed quantitative in situ approaches that integrate metaviromic sequencing with phage induction assays. In wastewater-impacted coastal zones, quantification of encapsidated ARGs within purified viral fractions reveals that transduction-competent ARGs can constitute up to 5–10% of the extracellular gene pool (Figure 2). Notably, mitomycin C-based prophage induction experiments using mixed microbial communities from outfall sediments have demonstrated that lysogenic conversion events can increase the transfer frequency of clinically relevant genes such as *bla*_CTX-M_ by an order of magnitude under nutrient-rich conditions. However, distinguishing generalized from specialized transduction events in metaviromic datasets without cultivation remains challenging, as short-read assembly often collapses genetic context. To circumvent this, long-read sequencing of viral DNA combined with host CRISPR spacer matching has been proposed as an emergent methodology to directly link phage-borne ARGs to specific donor hosts [26]. Methodologically, ex vivo phage-host interaction models utilizing fluorescently labeled ARG-containing phages and flow cytometry-sorted recipient cells now offer a tractable platform to measure transduction rates under manipulated environmental variables, such as temperature, salinity, and particle concentration. These models have shown that suspended particulate matter enhances phage-mediated transfer efficiency by concentrating phages and their bacterial hosts within localized microenvironments, effectively creating transduction micro-hotspots within outfall plumes. Despite these advances, the true ecological magnitude of transduction relative to conjugation in marine discharge environments remains unresolved, calling for systematic field surveys that quantify the per-cell transfer rates of both mechanisms simultaneously. Integrated frameworks that combine viromics, inducible phage diversity profiling, and controlled mesocosm experiments will be essential to close this critical knowledge gap in environmental gene flow dynamics (Figure 2).

## 5. From Indicator Organisms to Integrated Biological Monitoring

### 5.1. Beyond Traditional Fecal Indicator Bacteria

Traditional fecal indicator bacteria, such as *E*. *coli*, have long been used to monitor fecal contamination in marine environments, including direct sea discharge outlets. However, these indicators often suffer from limitations related to their ambiguous host specificity, which hampers accurate source tracking of pollution. Human-specific Bacteroides markers, notably HF183 and BacR287, have emerged as highly sensitive and specific molecular tools that overcome these limitations. These markers can precisely distinguish human fecal contamination from that of other animals, thereby providing a more accurate assessment of anthropogenic pollution in marine discharge areas. Their application in direct sea outfall monitoring enhances the resolution of source attribution, which is critical for targeted pollution control and public health risk assessment [23]. Moreover, pathogen-specific adaptive gene markers, such as the virulence-associated genes *toxR* and *tdh* in *Vibrio* species, serve as direct indicators of pathogenic risk without reliance on culture-based methods. This is particularly important because many pathogens enter a viable but non-culturable state in marine environments, leading to underestimation of their presence when using traditional cultivation techniques. Molecular detection of these virulence genes enables timely and accurate risk evaluation of pathogenic bacteria in discharge waters, facilitating proactive management strategies. The integration of human-specific Bacteroides markers with pathogen virulence gene detection thus represents a significant advancement beyond conventional fecal indicator bacteria (FIB), allowing for precise source identification and direct risk assessment of pathogenic microorganisms in direct sea discharge monitoring systems [63,64]. In a comparative study of 25 coastal outfall sites, human-specific *Bacteroides* markers (HF183) were detected in 92% of samples with observable sewage impact, at concentrations ranging from 3.6 × 10^3^ to 8.2 × 10^5^ copies/100 mL, while traditional FIB (*E. coli*) showed poor correlation (R^2^ = 0.21) with sewage proximity [23].

### 5.2. Microbiome-Level Index Construction

The complexity of microbial communities in marine discharge environments necessitates the development of integrative indices that quantitatively reflect ecological disturbances and associated risks. The microbiome deviance index (MDI) is a novel metric that quantifies the extent of microbial community deviation in discharge sites relative to reference pristine marine environments. By assessing shifts in community composition, MDI provides a biologically meaningful measure of ecological perturbation caused by pollution, offering an intuitive evaluation of contamination intensity. This index facilitates the detection of subtle microbial community changes that traditional chemical or indicator-based assessments might overlook, thereby enhancing environmental monitoring sensitivity [39]. The Microbiome Deviance Index (MDI) was calculated as the Bray–Curtis dissimilarity between impacted and reference sites, ranging from 0.12 (low impact) to 0.78 (high impact) across 12 discharge outlets, with a significant positive correlation (*ρ* = 0.83, *p* < 0.001) against pollutant load [39]. Complementing MDI, the antibiotic resistance risk index (ARRI) integrates multiple dimensions of ARG profiles, including their abundance, genetic mobility, and the pathogenic potential of host bacteria. This multidimensional approach enables a comprehensive risk assessment of the resistome in discharge environments, capturing not only the presence of ARGs but also their potential for horizontal gene transfer and clinical relevance. ARRI thus serves as a robust tool for quantitatively comparing antibiotic resistance risks across different discharge sites and temporal scales. Together, MDI and ARRI embody a microbiome-level paradigm for environmental risk assessment, providing holistic and quantitative frameworks that link microbial community dynamics with functional resistance traits, thereby informing pollution control and public health interventions in marine discharge contexts [40,65]. Despite their promise, the validation status and limitations of MDI and ARRI warrant critical consideration. MDI has been primarily validated in freshwater and soil systems [39]; its applicability to highly dynamic marine outfall environments with strong tidal mixing, variable salinity, and sediment resuspension requires further empirical testing. Reproducibility remains a challenge, as MDI values depend on the choice of reference “pristine” sites, sequencing platform, and bioinformatic pipeline [40]. Inter-laboratory comparisons and standardized reference datasets are urgently needed. Regarding ARRI, its weighting scheme for ARG mobility and host pathogenicity currently relies on expert knowledge and existing databases, which may introduce bias and fail to capture novel resistance determinants [40,65]. Moreover, ARRI does not yet incorporate gene expression data (metatranscriptomics), which is crucial for distinguishing latent risk from active resistance [46]. Despite these limitations, MDI and ARRI represent an important conceptual advance, shifting from binary indicator presence/absence to continuous, multidimensional risk indices. Future efforts should focus on prospective validation through longitudinal studies, establishment of threshold values for regulatory action, and integration with quantitative microbial risk assessment (QMRA) frameworks [41]. The Antibiotic Resistance Risk Index (ARRI) integrated three dimensions: ARG abundance (log_10_ copies/g), mobility potential (proportion of ARGs on MGEs, range: 12–45%), and pathogenicity (presence of ARGs in ESKAPEE pathogens). ARRI values ranged from 0.15 to 0.82 across sites, with the highest risk observed in sediments from shallow (depth < 5 m) and semi-enclosed outfall zones [40,65].

### 5.3. Real-Time Monitoring Potential of Environmental DNA

Environmental DNA technologies, combined with microfluidic chip platforms, have demonstrated promising capabilities for real-time monitoring of ARGs and pathogen markers in marine discharge outlets. Automated workflows encompassing sample collection, DNA extraction, and target gene amplification enable rapid quantification of ARGs and virulence gene markers within hours, significantly shortening the response time for pollution events. This rapid turnaround is critical for early warning systems and emergency response in coastal pollution management. However, several technical challenges currently limit the widespread application of eDNA-based real-time monitoring. Quantitative standardization remains a major bottleneck due to variable eDNA degradation rates in marine environments and the presence of PCR inhibitors, which can affect detection sensitivity and accuracy. Additionally, environmental background noise, including exogenous DNA contamination, can lead to false-positive signals, complicating data interpretation. Addressing these challenges requires the establishment of standardized protocols for eDNA sampling, processing, and quantification, as well as rigorous quality control frameworks to ensure data reliability. The development of industry-wide standards and validation procedures will be essential to fully harness the potential of eDNA-microfluidic technologies for continuous, in situ surveillance of microbial pollution and antibiotic resistance dissemination in marine discharge systems [40,66] (Table 2).

## 6. One Health Integration and Future Frontiers

### 6.1. Transmission Pathways from Direct Sea Outfalls to the Food Web

**Figure 3:**
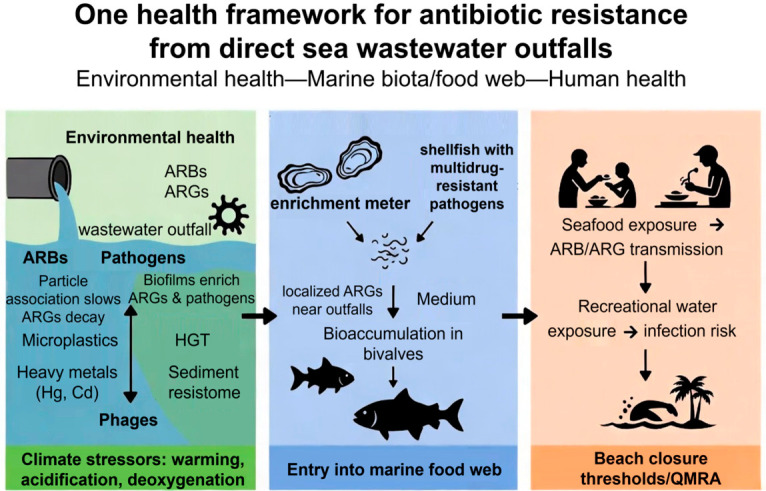
Schematic representation of the One Health framework applied to direct sea discharge outlets. Arrows indicate transmission pathways of antibiotic-resistant bacteria (ARB), antibiotic resistance genes (ARGs), and pathogens from outfalls into environmental compartments, marine food webs, and human exposure routes.

The dissemination of antibiotic-resistant bacteria and ARGs from direct sea wastewater outfalls into marine food webs represents a critical pathway for environmental and human health risks. One primary route involves the bioaccumulation of ARG-harboring bacteria in filter-feeding bivalves, which concentrate microorganisms from surrounding waters into their soft tissues. Studies have demonstrated that bivalves near wastewater discharge points exhibit significantly elevated levels of ARGs compared to those from offshore areas, indicating a localized enrichment of resistance determinants due to anthropogenic inputs [56]. Quantitative risk assessment models have further corroborated that the exposure risk to ARGs via seafood consumption is markedly higher in aquaculture zones adjacent to outfalls, underscoring the necessity for stringent food safety monitoring and regulatory oversight in these regions [56]. The presence of clinically relevant pathogens such as *P. aeruginosa* and *E. coli* carrying multidrug resistance genes in shellfish further amplifies the public health concern [56]. Beyond seafood consumption, direct human exposure occurs in recreational marine environments such as swimming beaches where contact with contaminated waters facilitates pathogen and antibiotic-resistant bacteria (ARB) transmission. The quantitative microbial risk assessment frameworks have been applied to estimate infection probabilities among swimmers exposed to wastewater-impacted coastal waters, providing data-driven thresholds for beach closures to mitigate infection risks [41]. These models integrate pathogen concentrations, exposure durations, and dose–response relationships to inform public health decisions. The persistence of ARB and ARGs in marine environments is influenced by factors such as particle association, which slows ARG decay and promotes horizontal gene transfer, thereby sustaining resistance reservoirs in coastal waters [27]. Moreover, biofilms on microplastics and suspended particles act as hotspots for ARG enrichment and pathogen colonization, further facilitating their entry into the marine food web [25]. Collectively, these findings highlight the complex transmission pathways of antibiotic resistance from direct sea outfalls through marine biota to humans, emphasizing the urgent need for integrated surveillance and risk management strategies encompassing environmental, food safety, and recreational exposure routes (Figure 3).

### 6.2. Virus–Bacteria Interactions

The interplay between bacteriophages and bacterial communities in aquatic environments is increasingly recognized as a pivotal factor modulating the structure of microbial populations and the dissemination of ARGs and VFs. Phage-mediated transduction, particularly via lysogenic cycles, enables the horizontal transfer of ARGs and VFs by packaging bacterial DNA and introducing it into new bacterial hosts upon infection. This mechanism accelerates the spread of resistance and virulence traits across diverse bacterial taxa, thereby influencing microbial community dynamics and resistance evolution [67]. Recent research has identified lysogenic phages carrying ARGs and VFs in wastewater-impacted environments, suggesting their role as mobile genetic element vectors that contribute to the resistome and virulome expansion [25]. However, quantifying the frequency and efficiency of phage transduction events in natural settings remains challenging due to methodological limitations. Current approaches rely on marker gene tracking and metagenomic analyses, but these often lack the resolution to definitively attribute gene transfer events to phages [26]. To address these gaps, emerging techniques combining viral metagenomics with bacterial metagenomics and experimental phage-host interaction assays are being developed. These integrative methods aim to elucidate the contribution of phage transduction to ARG and VFs dissemination quantitatively and mechanistically [68]. Understanding phage-bacteria interactions is crucial for predicting the evolution of antimicrobial resistance in marine and freshwater ecosystems impacted by wastewater discharge, as phages may either facilitate resistance spread or serve as biocontrol agents targeting pathogenic bacteria. Therefore, advancing molecular tools and experimental frameworks to dissect phage-mediated gene flow will enhance our capacity to manage microbial risks associated with environmental antibiotic resistance (Figure 3).

### 6.3. Artificial Intelligence and Digital Twins

The application of artificial intelligence and digital twin technologies offers transformative potential for monitoring and managing ARG dynamics and pathogenic bacteria abundance in wastewater discharge environments. Deep learning models, such as long short-term memory networks and transformer architectures, trained on extensive historical metagenomic datasets, can capture complex temporal patterns and environmental drivers influencing ARG and pathogen fluctuations. These models have demonstrated capability in predicting ARG abundance variations in response to environmental factors like rainfall and tidal cycles, enabling the establishment of dynamic pollution warning thresholds tailored to specific discharge sites [56]. Complementing predictive analytics, digital twin frameworks construct virtual replicas of direct sea outfall ecosystems by integrating microbial community data with physicochemical parameters and hydrodynamic models. Such coupled microbiome-environment simulations allow exploration of hypothetical scenarios, including varying wastewater pretreatment intensities, discharge depths, and diffuser designs, to forecast the evolution of resistomes under different management strategies [68]. This “virtual laboratory” approach facilitates informed decision-making by evaluating the efficacy of interventions in silico prior to implementation, thereby optimizing resource allocation and minimizing ecological impacts. Moreover, the AI-driven analyses can identify key microbial taxa and resistance determinants serving as early indicators of pollution events, enhancing real-time surveillance capabilities [69]. Despite these advances, challenges remain in integrating heterogeneous data sources, ensuring model interpretability, and scaling digital twins for complex coastal systems. Continued development of AI methodologies tailored to environmental microbiology, coupled with high-resolution monitoring infrastructures, will be essential to realize the full potential of these technologies in mitigating antibiotic resistance dissemination from marine wastewater outfalls.

### 6.4. Unresolved Issues

Several critical knowledge gaps persist in the effective monitoring and risk assessment of antibiotic-resistant pathogens in marine environments impacted by wastewater discharge. One major challenge is the detection and quantification of the viable but non-culturable (VBNC) pathogenic bacteria, such as *Vibrio vulnificus* and *E. coli* O157:H7, which evade conventional culture-based methods yet retain infectivity and resistance potential. Current detection techniques lack sensitivity and specificity for VBNC cells, necessitating the development of advanced molecular assays targeting active cellular RNA markers or metabolic activity probes to accurately assess their presence and viability in environmental samples [43]. Another unresolved issue concerns the so-called antibiotic resistance “dark matter” in sediments, ARGs that remain unannotated due to limited database representation and unknown genetic contexts. These cryptic ARGs may be mobilized and transferred to active bacterial populations following environmental disturbances such as dredging or storm events, posing unpredictable risks for resistance proliferation [42,70]. The mechanisms governing the release, horizontal gene transfer, and ecological impacts of these sediment-associated ARGs under dynamic environmental conditions remain poorly understood. Addressing these uncertainties requires integrative approaches combining metagenomics, functional genomics, and environmental perturbation experiments to elucidate the mobilization potential and risk profiles of sediment resistomes. Furthermore, the interactions between ARGs and co-selective agents like heavy metals and microplastics in sediments add complexity to resistance dynamics and demand comprehensive investigation [25]. Resolving these unresolved issues is imperative for refining environmental surveillance paradigms and developing robust, predictive frameworks for antibiotic resistance risk management in marine ecosystems influenced by wastewater discharge (Figure 3). Beyond VBNC pathogens and resistance “dark matter,” several additional unresolved issues warrant attention. First, the quantitative contribution of phage-mediated transduction versus conjugation to ARG dissemination in marine outfalls remains unknown, necessitating field studies that simultaneously measure per-cell transfer rates of both mechanisms [37,38]. Second, the interactive effects of climate change stressors (warming, acidification, deoxygenation) on HGT frequency and virulence expression are poorly characterized [22,55]. Third, current risk assessment frameworks rarely incorporate the spatiotemporal heterogeneity of ARG hotspots, leading to potential underestimation of exposure risks [25,26]. Addressing these gaps will require multi-site, long-term observatories, standardized protocols for eDNA and viability assays, and next-generation predictive models that integrate environmental, ecological, and evolutionary processes [68,69].

## 7. Conclusions

The shift from taxonomic inventories to a systems-level approach integrating metagenomics and multi-omics has transformed monitoring of direct sea discharge outlets. This review has synthesized current knowledge demonstrating that antibiotic resistance genes and virulence factors are frequently coupled via horizontal gene transfer, creating compounded microbial risks. Co-selective pressures from heavy metals and biocides amplify this coupling, while the mobilome (integrons, plasmids, phages) serves as the central genetic conduit. Traditional fecal indicator bacteria are inadequate to capture these complexities; instead, microbiome-level indices such as MDI and ARRI provide holistic, risk-based frameworks. We advocate for integrating metagenomic ARG analysis into regulatory monitoring, redefining outfalls from pollution endpoints to sentinel sites for early risk detection.

Looking forward, several priority actions emerge. Regulatory frameworks at national and international levels, such as the EU Marine Strategy Framework Directive, should consider incorporating metagenomic ARG profiling and indices like MDI and ARRI as complementary indicators, supported by pilot studies to establish baseline thresholds. Methodological standardization is urgently needed, including ISO-level protocols for eDNA sampling, ddPCR quantification, and bioinformatic analysis of resistome data to ensure cross-study comparability and reproducibility. Research priorities should focus on the detection and risk assessment of viable but non-culturable pathogens using RNA-based or metabolic activity probes, the characterization of resistance “dark matter” in sediments through functional metagenomics and long-read sequencing, and the quantification of phage-mediated transduction in situ. Technology adoption must be accelerated, particularly the investment in artificial intelligence and digital twin models trained on multi-year metagenomic and environmental datasets to enable predictive early warning systems for ARG hotspots. Finally, One Health collaboration should be strengthened through international networks that link clinical, veterinary, and environmental surveillance to trace ARG transmission pathways from outfalls to humans, enabling source attribution and targeted interventions.

In conclusion, metagenomics and multi-omics integration in direct sea discharge monitoring enable a proactive, anticipatory risk management paradigm. Addressing the unresolved challenges of VBNC pathogens, resistance dark matter, and phage transduction, while advancing regulatory frameworks and emerging technologies (digital twins, eDNA microfluidics), will be essential for safeguarding coastal ecosystems and public health in an era of increasing urbanization and climate change.

## Figures and Tables

**Table 1:** Representative studies on bacterial communities, antibiotic resistance genes (ARGs), and virulence factors (VFs) in direct sea discharge outlet environments.

Environmental Sample	Location	Analytical Methods	Key Bacterial Taxa Enriched	Key ARGs/VFs Detected	Major Findings	Reference
Sediment and seawater	Bay of Bengal, India	16S rRNA amplicon sequencing	*Vibrio*, *Pseudomonas*, *Aeromonas*	NA	Moderate pollution peaks diversity; temperature/salinity modulates pathogens	[51]
Estuarine sediment	South China Sea (Dapeng Cove)	Metagenomics, qPCR	Proteobacteria, Bacteroidetes	*sul1*, *tet(A)*, *bla*_CTX-M_, *intI1*	ARGs co-occur with integrase genes; HGT hotspots	[21]
Coastal seawater	Central Adriatic Sea	Long-read metagenomics (Nanopore)	Enterobacterales	*bla*_KPC_, *bla*_TEM_ on plasmids	Complete plasmid assembly reveals ARG-VF co-localization	[45]
Microplastics from effluent	South Africa	16S rRNA, qPCR	*E. coli*, *Klebsiella*, *Enterococcus*	Carbapenem resistance genes	Microplastics act as ARG reservoirs	[24]
Oyster gut	Urbanized estuary, USA	Metatranscriptomics	Stable taxa	Nutrient cycling genes expressed	Functional plasticity without taxonomic shift	[46]
Sediment and water	Pearl River Estuary, China	Null model, NCM	Abundance and rare taxa	Heavy metal resistance genes	Deterministic selection dominates abundant taxa	[34]
Shellfish near outfall	Multiple sites (UK/Finland)	High-throughput qPCR	*P. aeruginosa*, *E. coli*	Multidrug resistance genes	Higher ARG burden in bivalves near outfalls	[56]

NA: Not specified.

**Table 2:** Comparison of traditional monitoring tools versus emerging microbiome-based indicators for direct sea discharge outlets.

Criterion	Traditional Tools (FIB, Physicochemical)	Emerging Microbiome-Based Indicators	References
Information provided	Presence of fecal contamination, bulk chemical status	Taxonomic composition, functional genes (ARGs, VFs), mobility potential	[45,52]
Source attribution capability	Poor	High	[50,53]
Detection of VBNC pathogens	No (culture-dependent underestimation)	Yes (molecular detection of virulence genes)	[48,51]
Assessment of ARG/VF risks	No	Yes (ARRI, MDI, resistome profiling)	[39,40]
Throughput	Low to medium	High	[56]
Cost per sample	Low ($5–20)	Medium to high ($50–500 depending on method)	[44,45]
Real-time potential	Yes (sensors for temp, pH, nutrients)	Emerging (eDNA + microfluidics, but standardization needed)	[17,47]
Regulatory status	Established	Experimental; not yet adopted in routine monitoring	[17,47]

## Data Availability

No new data were created or analyzed in this study. Data sharing is not applicable to this article.
